# Prevalence of methicillin resistant coagulase negative staphylococci in a tertiary care hospital

**Published:** 2010-12

**Authors:** V Sharma, N Jindal, P Devi

**Affiliations:** Department of Microbiology, Government Medical College, Amritsar 143001, Punjab, India.

**Keywords:** Coagulase negative Staphylococci, minimum inhibitory concentration, E test

## Abstract

**Background and Objectives:**

Presence of methicillin and multidrug resistance has associated coagulase negative staphylococci (CNS) with high morbidity and mortality worldwide. The present study was carried out to study the susceptibility pattern of CNS to various antimicrobial agents and to determine the prevalence of CNS methicillin resistance in our hospital setting.

**Materials and Methods:**

A total of 300 strains of CNS isolated from various clinical specimens were subjected to speciation and their antimicrobial sensitivity testing was studied by Kirby Bauer disc diffusion method. Methicillin resistance was studied by observing minimum inhibitory concentration (MIC) of Oxacillin by macrobroth dilution method and E test. Susceptibility to vancomycin was determined by vancomycin screen agar test and minimum inhibitory concentration by macrobroth dilution test.

**Results:**

All the isolates were susceptible to vancomycin, linezolid and teicoplanin in disc diffusion test while maximum resistance was noted against penicillin (100%) followed by ciprofloxacin (36.3%), norfloxacin (34.3%), gentamicin (34%), nitrofurantoin (29.9%), erythromycin (27.9%) and amikacin (22.7%). Fifty two percent (n = 156) of the isolates were found to be resistant to methicillin. A comparison between resistance patterns of methicilin resistant and methicillin sensitive strains showed that methicillin resistant isolates had higher level of resistance to other antibiotics.

**Conclusions:**

The high level of resistance among CNS to commonly used antimicrobial agents in our hospital is a matter of great concern and can be prevented by practices of effective infection control measures.

## INTRODUCTION

Though a part of normal cutaneous ecosystem, coagulase negative staphylococci (CNS) have assumed a great pathogenic potential and are being recognized as important agents of nosocomial infection (1). These organisms express resistance to multiple antibiotics which not only pose a serious therapeutic problem but also serve as a hospital reservoir of antibiotic resistance genes. An increase in incidence of nosocomial infections caused by CNS which are resistant to methicillin (MRCNS) and other antibiotics has been reported in some of the studies (2, 3). This prospective study was therefore undertaken to determine currently prevalent antibiogram of coagulase negative staphylococci isolated from various clinical specimens obtained from inpatients of a tertiary care hospital.

## MATERIALS AND METHODS

A total of 300 CNS strains, isolated from various clinical specimens between January 2008 and December 2009, were processed in Department of Microbiology, Government Medical College in Amritsar. The strains selected in this study had the potential of being clinically significant on the basis of source and quantity and infective agent or both. This included the isolates from blood, pus (sole organism or in moderate to heavy amounts), urine (Greater than 105 organisms per ml), intravenous catheter tips (greater than 15 colonies), ascetic fluid and synovial fluid. All the blood cultures were from long term admitted or seriously compromised patients with indwelling intravenous catheters. All the isolates were identified by standard procedures (Gram staining, catalase test, slide and tube coagulase test) and were subjected to speciation. The tests, which were simple, inexpensive and easy to perform, were selected from the scheme of Kloos and Schleifer (1).

All the 300 isolates were subjected to antibiotic sensitivity testing by Kirby Bauer Disc diffusion method (4). The antimicrobial agents used were penicillin (10 IU/disc), erythromycin (15 µg/disc), ciprofloxacin (5 µg/disc), norfloxacin (10 µg/disc), nitrofurantoin (300 µg/disc), gentamicin (10 µg/disc), amikacin (30 µg/disc), vancomycin (30 µg/disc), teicoplanin (30 µg/disc) and linezolid (30 µg/disc) (4).

Methicillin resistance was studied by observing minimum inhibitory concentration of oxacillin by acrobroth diluition method and E (epsilometer) test (Hi Comb) (4, 5).

**Oxacillin MIC test**. Serial dilutions of oxacillin (0.25–256 µg/ml) were prepared in Muller Hinton broth (Hi Media, Mumbai, India) with added 2% NaCl. The inoculum was prepared by diluting 0.5 McFarland suspension to the concentration of 105 CFU/ml. The tubes were inoculated and incubated at 35°C for 24 hours. All isolates with MIC of ≥0.5 µg/ml were taken as resistant and ≤0.25 µg/ml as sensitive. Two tubes, one growth control tube with no oxacillin, and one sterility control tube with no inoculum were also incorporated in the tests. *Staphylococcus aureus* ATCC 29213, was used as a reference strain (4, 5).

**E Test for oxacillin**. The E-test (Hi Comb) was performed as per manufacturer's instructions. An ellip- tical zone of inhibition was obtained after incubation and MIC was read where ellipse intersected the strip.

**Determination of MIC for vancomycin**. Suscepti- bility to vancomycin was determined by broth dilution method. Serial dilutions of vancomycin (0.5–128 µg/ ml) were prepared in Muller Hinton broth (Hi media, Mumbai). The inoculum was prepared by diluting 0.5 McFarland suspension to the concentration of 10^5^ CFU/ml. The tubes were inoculated and incubated at 35°C for 24 hours. *Staphylococcu, aureus*, ATCC 29213 and *Entercoccus faecalis*, ATCC 29212, were used as vancomycin susceptible controls and *E. faecalis* 51299 as vancomycin resistant control. Readings were taken as per the guidelines of CLSI. Isolates with MIC ≤2 µg/ml were taken as susceptible, MIC=4–8 µg/ml as intermediate, and MIC ≥16µg/ ml as resistant (4–6).

**Vancomycin screen agar test**. Brain heart infusion agar (Hi Media, Mumbai) plates containing 6µg/ml of vancomycin were used for vancomycin screen agar. The plates were inoculated with the test strain from the standard inoculum and incubated at 35°C for 24 hours. Any visible growth indicated resistance. *S. aureus*, ATCC 29213, was used as a reference strain (4, 6).

## RESULTS

Of 300 strains of CNS studied, 139 (46.33%) were isolated from blood followed by 73 from pus (24.33%), 67 (22.33%) from urine, 17 (5.7%) from catheter tips and 2 (0.6%) each from ascetic and synovial fluids. *S. epidermidis* was the predominant species (n=162, 54%) and was most frequently isolated from all the specimens except urine. The second most common species was *S. hemolyticus* (n=120, 40%) and its commonest source of isolation was urine (n = 50, 41.66%). The other species of CNS identified were *S. warneri* (n=8, 2.67%), *S. saprophyticus* (n=7, 2.33%) and *S. cohnii* (n=3, 1.10%).

All 300 isolates were found to be uniformly susceptible to linezolid, vancomycin and teicoplanin. Resistance to penicillin was 100% and was followed by that of ciprofloxacin (36.3%), norfloxacin (34.3%), gentamicin (34%), nitrofurantoin (29.9%), erythromycin (27.9%) and amikacin (22.7%). Study of Oxacillin MIC showed that of the 300 strains, 144 (48%) were sensitive to oxacillin with MIC ≤0.25 µg/ml and 156 (52%) were resistant with MIC≥0.5 µg/ml. MIC of 156 resistant isolates ranged between 0.5 µg/ml to 32 µg/ml. Methicillin resistant CNS (MRCNS) showed higher level of resistance to other antimicrobial agents studied as compared to methicillin sensitive CNS (MSCNS) (Table 1). All the study isolates failed to grow on vancomycin screen agar and their MIC value for vancomycin was < 2 µg/ml in broth macrodilution test.

**Table 1:** Statistical analysis of antibiotic resistance pattern of MRCNS and MSCNS.

**Antibiotics**	**MRCNS % Resistance**	**MSCNS % Resistance**	**p value**	**Statistical significance**
Penicillin	100	100	–	–
Ciprofloxacin	47.6	24.1	<0.05	Significant
Gentamicin	45.3	21.6	<0.05	Significant
Norfloxacin*	44.8	27.5	>0.05	Non significant
Nitrofurantoin*	41.3	20.6	>0.05	Non significant
Erythromycin#	31.6	23	>0.05	Non significant
Amikacin	31.5	13	<0.05	Significant
Vancomycin	0	0	–	–
Linezolid	0	0	–	–

* =used only in urine isolates

# = not used in urine isolates

MRCNS=Methicillin resistant coagulase negative staphylococci

MSCNS=Methicillin sensitive coagulase negative staphylococci

## DISCUSSION

Our study identified five different species/groups of CNS namely *S. epidermidis*, *S. haemolyticus*, *S. saprophyticus*, *S. warneri* and *S. cohnii*. *S. epidermidis* was the commonest (54%) followed by *S. haemolyticus* (40%) which was also the most frequent isolate from blood cultures and intravenous catheter tips which is similar to the findings of other authors and signifies its importance as an agent of nosocomial bacteremia and catheter related sepsis (1, 7, 8). *S. haemolyticus* was the predominant isolate from urine specimens in our study (50 of 67, 74.62%). In the study of Chaudary et al**., it's isolation rate from urine specimens was 84.1%. Other studies have also reported this species as an important pathogen of nosocomial urinary tract infections (9).

Antibiotic resistant CNS has emerged as a major cause of morbidity and mortality in hospital setting during the last decade. Majority of CNS recovered in our study also showed multidrug resistance but the susceptibility pattern was at variance with that reported in some other studies. Many studies from different parts of the world have reported presence of multidrug resistance in CNS (10, 11). Also from India, many authors have reported multidrug resistance in coagulase negative staphylococci (1, 7, 12, 13). Patricia et al. have reported 61% of their study isolates to be multidrug resistant (14). This could be because of different protocols and panels of antibiotics used in various hospitals and perhaps also due to differential clonal expression and drug pressure in the community (15).

In the present study, the prevalence of methicillin resistance among CNS in our hospital was 52% which is less than that reported from Delhi (62.7%) but more than that from Lucknow (38%) (7, 16). In an earlier study (1997–98) conducted in our hospital, CNS showed 20.8% methicillin resistance (15). This increase in incidence of MRCNS in our hospital in last one decade is statistically significant and has become a potential problem.

A comparison between antibiotics sensitivity patterns of MRCNS and MSCNS of our study revealed that MRCNS had higher level of resistance to many antimicrobials as compared to MSCNS. Similar findings showing higher antibiotic resistance among MRCNS have been reported by many authors (7). The difference was statistically significant for ciprofloxacin, gentamicin and amikacin (p<0.05). Oliveria et al. found this difference statistically significant for ciprofloxacin and gentamicin (p≤0.05) (17).

All the MRCNS in our study were susceptible to glycopeptides (vancomycin and teicoplanin) which corroborates the findings of many authors (7, 8, 10, 12, 15, 16). However, there are reports of decreased susceptibility to frank resistance to vancomycin and teicoplanin from our country (6, 9, 18). One hundred percent susceptibility of strains isolated our study to glycopeptides suggests prudent use and continuous monitoring of MIC levels so that we may not fall back into pre-antibiotic era.

It is thus concluded that isolation of CNS and their antibiotic susceptibility pattern should be regarded with all seriousness in clinical practice and clinical epidemiology because by being resistant to multiple antibiotics (methicillin resistant CNS, in particular), their prevalence not only limits the treatment options but also act as a reservoir of drug resistance genes. MRCNS are emerging nosocomial pathogens and every effort should be made for prevention and control of infections caused by MRCNS which depends on practices of effective hospital infection control measures and minimization of risk factors by doing regular surveys of health care providers to detect and treat the CNS carriers so that they do not transmit the CNS to the patients.

## References

[1] Washington WC, Allen SD, Jand WM, Koneman W, Procop G, Schreckenberger PC (2001). Gram Positive Cocci- part1. Koneman's Color Atlas and Textbook of Diagnostic Microbiology.

[2] Gahrn-Hansen B (1987). Coagulase negative staphylococci and micrococci in clinical microbiology. Dan Med Bull.

[3] Kloss WE, Bannerman TL (1994). Update on clinical 3. significance of coagulase negative staphylococci. Clin Microbiol Rev.

[4] Wayne PA (2005). Clinical and Laboratory standards institute 2005.

[5] Miles RS, Amyes SGB, Collee JG, Fraser AG, Marmion BP, Simmons A (1996). Laboratory control of antimicrobial therapy. Mackie and McCartney Practical Medical Microbiology.

[6] Tiwari HK, Sen MR (2006). Emergence of vancomycin resistant *Staphylococcus aureus* (VRSA) from a tertiary care hospital from northern part of India. BMC Infect Dis.

[7] Singhal R, Dhawan S, Mohanty S, Sood S, Kapil A, Dhawan B (2006). Species distribution and antimicrobial susceptibility of coagulase negative staphylococci in a tertiary care hospital. Ind J Med Res.

[8] Gill VJ, Selepak ST, Williams EC (1983). Species identification and antibiotic susceptibility of coagulase negative staphylococci isolated from clinical specimens. J Clin Microbiol.

[9] Chaudhury A, Kumar AG (2007). In vitro activity of antimicrobial agents against oxacillin resistant staphylococci with special reference to *Staphylococcus haemolyticus*. Ind J Med Microbiol.

[10] Udo EE, Jacob LE, Chugh TD (2009). Antimicrobial resistance of coagulase negative staphylococci from a Kuwait hospital. Microb Drug Resist.

[11] Marsik FJ, Brake Sylvia (1982). Species identification and susceptibility to 17 antibiotics of coagulase negative staphylococci isolated from clinical specimens. J Clin Microbiol.

[12] Goyal R, Singh NP, Kumar A, Kaur I, Singh M, Sumita N (2006). Simple and economical methods of speciation and resistotyping of clinically significant coagulase negative staphylococci. Ind J Med Microbiol.

[13] Shrikhande S, Thakar YS, Pathak AA, Saoji AM (1996). Species distribution of clinical isolates of staphylococci. Ind J Pathol Microbiol.

[14] Patricia AK, Shashikala, Sheela DC, Srinivasan S, Reba Kanungo (2007). Profile of coagulase negative staphylococci associated with infections in a tertiary care hospital. Biomed.

[15] Mohan V, Jindal N, Aggarwal P (2002). Species distribution and antibiotic sensitivity pattern of coagulase negative staphylococci isolated from various clinical specimens. Ind J Med Microbiol ; 20: 45-46. Microbiol Rev1994.

[16] Singh RK Simple method for speciation of clinically significant coagulase negative staphylococci and its antibiotic sensitivity/resistant pattern in nicu of tertiary care centre. Ind Medica.

[17] Oliveria ADD, Azevedoz PA, Sousa LB, Niero CV, Lottenberg C, Martino MDV (2007). Laboratory detection methods for methicillin resistance in coagulase negative staphylococcus isolated from ophthalmic infections. Arq Bras Oftalmol.

[18] Menesez GA, Harish BN, Sujatha S, Vinathini K, Parija SC (2008). Emergence of vancomycin intermediate staphylococcus species in southern India. J Clin Microbiol.

